# Profile of gynecological cancers in a tertiary hospital, Eastern Nigeria

**DOI:** 10.11604/pamj.2023.44.139.39034

**Published:** 2023-03-17

**Authors:** Emily Akuabia Nzeribe, Nathan Azubuike Ododo, Precious Okechukwu Eteike

**Affiliations:** 1 Gynaecological Oncology Unit, Department of Obstetrics and Gynaecology, Federal University Teaching Hospital Owerri, Imo State, Nigeria

**Keywords:** Profile, gynecological, cancers, retrospective, cross-sectional, study, Nigeria

## Abstract

**Introduction:**

there is a great diversity in the profile of cancers in the world. This study set out to analyze the profile of gynecological cancer in Federal University Teaching Hospital, Owerri, [FUTHO] (former Federal Medical Centre, Owerri, Imo state, Nigeria). Methods: this was a retrospective cross sectional descriptive study of the records of women admitted in the gynecological ward in FUTHO from January 2020 to November 2022. It was analyzed using SPSS version 23.0 and reported in simple percentages for categorical variables and measures of central tendency for quantitative variables.

**Results:**

a total of 1,378 gynecological patients were admitted into the Gynaecological ward of the hospital, out of which 242 (17.6%) were cancer cases. The most common cancer over the three years in review, was ovarian, 81 (33.5%), followed by cervical, 66 (27.3%), endometrial, 65 (26.8%), choriocarcinoma, 22 (9.1%), vulvar, 6 (2.5%) and vagina, 2 (0.8%). The most common gynecological cancers in this study is very different from previous reports from Nigeria and other African countries. The pattern looks like that seen in developed countries where endometrial and ovarian cancers top the list.

**Conclusion:**

this report shows a possible change in lifestyle and improved access to cervical cancer prevention strategies. It is also assumed that all the facilities who have recorded cervical cancer as the most common cancer can actually have a similar result as ours if a more current review is done.

## Introduction

Gynecological cancers occur in the female reproductive system such as the vulva, vagina, cervix, uterus, fallopian tubes, ovaries and peritoneum. They are named after the organ of origin [1]. Despite the drastic changes in the epidemiological profile of the gynecological cancers in the world space with very recent advances in the management of malignancies, gynecological cancers remain a major public health concern in Low and Middle-income Countries. Globally the International Agency for Research on Cancer reported that gynecological cancer contributed 19% of 5.1 million estimated new cancer cases and 2.9 million cancer deaths [2]. Cervical cancer contributed 0.6 million new cases in 2020, resulting in 342,000 deaths worldwide [3]. There is a great diversity in the profile of cancers in the world. The low and middle-income countries have a different profile possibly because of underreporting. In the Low and Middle-income countries, lack of awareness, late presentation, paucity of manpower as well as absence of recent innovations in the diagnosis, treatment and follow-up of malignancies have resulted in the discrepancies. Many years back, cervical cancer was the most common cancer of the female genital tract but with the advent of screening using the Papanicolaou test and other methods, the incidence of cervical cancer has drastically dropped globally.

The incidence of ovarian and endometrial cancer has gradually topped the statistics. This has resulted in a sharp difference and it was noticed that cervical cancer is no longer of prime issue in the world. Cervical cancer, despite being on a declining trend remains the second commonest cancer in Indian women second only to breast [4]. A report from Enugu, Nigeria showed that between 2000 and 2009, cervical cancer was the commonest gynecological cancer [5]. In Korle Bu Teaching Hospital, Ghana, the commonest gynaecological cancers were cervical cancer (57.8%) followed by ovarian cancer, endometrial cancer, choriocarcinoma and vulval carcinoma [6]. Similarly, in Nepal, 85.23 % of cases were cervical cancer, followed by ovarian cancer (3.16%, vulva (2.7%) vagina (2.11%), endometrium 0.33% and choriocarcinoma (0.07%) [7].

On the contrary, in some countries like Pakistan, cervical cancer was not the commonest gynecological malignancy with ovarian tumors leading with 42.4% of all gynecological cancers [8]. In a Polish National Cancer Registry data (1980-2018), a significant decrease in cervical and vaginal cancers was noticed with an increase in ovarian and corpus uteri cancers [9]. There is a distinct margin of difference in the pattern of gynecological cancers based on the resource status of the countries. Frequency of gynecological cancers varies from as low as 12.7-13.4% in North America and other developed nations to as high as 31.6-35.0% in sub-Saharan Africa. This is due to the difference in health-seeking behavior and availability of management options [10]. This study set out to determine the pattern of gynecological cancer in Federal Medical Centre, (FMC) Owerri, Imo state, Nigeria, (now Federal University Teaching Hospital, Owerri, FUTHO). Knowing that the burden of any disease in any geographical location is an important tool in the development of policies and strategies for disease prevention and control for that location, there is need for continued review of data on the pattern of gynecological cancers especially as preventive and diagnostic measures get improved albeit slowly in Nigeria [7].

## Methods

**Study design**: this was a retrospective cross-sectional descriptive study. The records of admission over this three-year period were reviewed and the frequency of the different cancers in the centre, the age at presentation and other important data were collected using a proforma.

**Study setting**: the study was carried out at the gynecological ward of the Federal University Teaching Hospital, Owerri, (FUTHO). FUTHO is a tertiary hospital in Imo State and a major reference centre in the southeastern and southsouthern parts of Nigeria which includes parts of Abia, Anambra, Rivers states and environs.

**Study participants**: these were women admitted into the Gynaecological ward and were on management for Gynaecological cancer between January 2020 and November 2022.

**Data analysis**: data were analyzed using (SPSS) Statistical Package for the Social Sciences (SPSS) software program, version 23.0 (IBM Corp., Armonk N.Y., USA). This was reported in simple percentages for categorical variables and measures of central tendency for quantitative variables. Significant association between categorical variables were tested using chi-square test and p< 0.05 was considered statistically significant.

**Ethical clearance**: ethical approval was obtained from the Ethical Review Board of the Federal University Teaching Hospital Owerri.

## Results

During the period of review (2020-2022), a total of 1,378 gynaecological patients were admitted into the Gynaecological ward of the Federal Medical Centre, Owerri, now Federal University Teaching Hospital, Owerri (FUTHO) out of which 242 (17.6%) were cancer cases. The ages of the women affected by these cancers during the periods of review ranged from 18-92 years. Generally, and according to the years in review, the median age affected by these cancers was 58 years (18-92), 58 years (24-75) and 50 years (33-76) in 2020, 2021 and 2022 respectively. The youngest cancer patients were as young as 18 years of age and had ovarian cancers while the oldest had cervical, vulval and vaginal cancers which occurred between 82 and 92 years (Table 1, Table 2 and Table 3). Specifically, the median age affected by these malignancies during the years in review was 55 years for cervical cancer (ranged 24-85); 54 years for ovarian cancer (ranged 18-76); 62 years for endometrial cancer (ranged 33 -78); 41 years for choriocarcinoma (ranged 26-49); 53 years for vulva cancer (ranged 42-92) and 68 years for vagina cancer (ranged 67-69).

**Table 1:** age distribution of different cancer patients in 2020

Year of Presentation	Age Group	Cervix n (%)	Ovary	Endometrium	Choriocarcinoma	Vulva	Vagina	Total
**2020**	< 20	0(0.0)	2(11.8)	0(0.0)	0(0.0)	0(0.0)	0(0.0)	2(3.2)
	20-29	3(11.5)	1(5.9)	0(0.0)	2(100.0)	0(0.0)	0(0.0)	6(9.5)
	30-39	2(7.7)	0(0.0)	1(7.7)	0(0.0)	0(0.0)	0(0.0)	3(4.8)
	40-49	6(23.1)	5(29.4)	1(0.0)	0(0.0)	0(0.0)	2(66.4)	13(20.6)
	50-59	2(7.7)	4(23.5)	2(15.4)	0(0.0)	0(0.0)	0(0.0)	8(12.7)
	60-69	11(42.3)	3(17.6)	2 (46.2)	0(0.0)	0(0.0)	1(33.3)	21(33.3)
	0-79	1(3.8)	2(11.8)	4(30.8)	0(0.0)	0(0.0)	0(0.0)	7(11.1)
	80-89	1(3.8)	0(0.0)	0(0.0)	0(0.0)	1(25.0)	0(0.0)	2(3.2)
	90 – 99	0(0.0)	0(0.0)	0(0.0)	0(0.0)	1(25.0)	0(0.0)	1(1.6)
	Total	26(100.0)	17(100.0)	13(100.0)	2(100.0)	2(100.0)	3(100.0)	63(100.0)

**Table 2:** age distribution of different cancer patients in 2021

Year of Presentation	Age Group	Cervix n(%)	Ovary	Endometrium	Choriocarcinoma	Vulva	Vagina	Total
2021	<20	0(0.0)	0(0.0)	0(0.0)	0(0.0)	0(0.0)	0(0.0)	0(0.0)
	20-29	1(5.9)	1(3.0)	1(3.2)	1(10.0)	0(0.0)	0(0.0)	4(4.3)
	30-39	3(17.6)	2(6.1)	0(0.0)	1(10.0)	0(0.0)	0(0.0)	6(6.5)
	40-49	3(17.6)	3(9.1)	3(9.7)	8(80.0)	1(100.0)	0(0.0)	18(19.6)
	50-59	5(29.4)	14(42.4)	8(25.8)	0(0.0)	0(0.0)	0(0.0)	27(29.3)
	60-69	4(23.5)	10(30.0)	17(54.8)	0(0.0)	0(0.0)	0(0.0)	31(33.7)
	70-79	1(5.9)	3(9.1)	2(6.5)	0(0.0)	0(0.0)	0(0.0)	6(6.5)
	80-89	0(0.0)	0(0.0)	0(0.0)	0(0.0)	0(0.0)	0(0.0)	0 (0.0)
	90 – 99	0(0.0)	0 (0.0)	0(0.0)	0(0.0)	0 (0.0)	0(0.0)	0(0.0)
	**Total**	17(100.0)	33(100.0)	31(100.0)	10(100.0)	1(100.0)	0(0.0)	92 (100.0)

**Table 3:** age distribution of different cancer patients in 2022

Year of Presentation	Age Group	Cervix n(%)	Ovary	Endometrium	Choriocarcinoma	Vulva	Vagina	Total
2022	< 20	1 (4.3)	0(0.0)	0(0.0)	0(0.0)	0(0.0)	0(0.0)	1(1.1)
	20- 29	0(0.0)	0(0.0)	0(0.0)	0(0.0)	0(0.0)	0(0.0)	0(0.0)
	30-39	2(8.7)	7(22.6)	3(14.3)	0(0.0)	0(0.0)	0(0.0)	12(13.8)
	40- 49	6(26.1)	13(41.9)	2(9.5)	10(100.0	0(0.0)	0(0.0)	31(35.6)
	50-59	4(17.4)	9(29.0)	6(28.6)	0(0.0)	0(0.0)	0(0.0)	19(21.8)
	60-69	(43.5)	(6.5)	(38.1)	0(0.0)	(100.0)	(100.0)	(25.3)
	70-79	0(0.0)	0(0.0)	2(9.5)	0(0.0)	0(0.0)	0(0.0)	2(2.3)
	80-89	0(0.0)	0 (0.0)	0(0.0)	0(0.0)	0(0.0)	0 (0.0)	0 (0.0)
	90 – 99	0(0.0)	0(0.0)	0(0.0)	0(0.0)	0 (0.0)	0(0.0)	0(0.0)
	**Total**	23(100.0)	31(100.0)	21(100.0)	10(100.0)	1(100.0)	1(100.0)	87(100.0)

In 2020 and 2021, majority of cancer, 21 (33.3%) and 31 (33.7%) cases respectively occurred in the 60 to 69 age group but this decreased to the 40-49 age group, 31 (35.6%) in 2022 (Table 1, Table 2 and Table 3). Out of the 242 cancer cases admitted, 104 (43.0 %) were old cases while 138 (57.0 %) were new cases (Table 4). Over the years, the new cases were 49 (77.8%), 47 (48.9%) and 44(50.6%) in 2020, 2021 and 2022 respectively. The differences in the total number of new cases during the periods of review were significant (P=0.021). The most common cancer (both old and new cases) over the three years in review (2020-2022) was ovarian, 81 (33.5%), followed by cervical, 66 (27.3%), endometrial, 65 (26.8%), choriocarcinoma, 22 (9.1%), vulvar, 6 (2.5%) and vagina, 2 (0.8%). However for individual years in review, in 2020, the most common cancer was cancer of the cervix, 26 (41.3%), followed by ovarian, 17 (27.0%), endometrial 13 (20.6 %), vulvar 4 (6.3%), choriocarcinoma, 2 (3.2 %), and vagina cancer, 1 (1.6%) while in 2021, cancer of the ovary topped the list, 33 (35.9%), followed by endometrium, 31(33.7%), cervical, 17 (18.5%), choriocarcinoma, 10 (10.9%), vulva, 1 (1.1%) and vagina nil. In 2022, ranking top was cancer of the ovary, 31 (35.6%), and then cervical, 23 (26.4%), endometrial 21 (24.1%), choriocarcinoma, 10(10.5 %), vulva and vagina, 1 (1.1 %) each (Table 4).

**Table 4:** distribution pattern of the old and new cancer cases (2020 to 2022)

		Diagnosis						
		Cervix	Ovary	Endometrium	Choriocarcinoma	Vulva	Vagina	Total
2020	Old	7(50.0)	4(28.6)	2(14.3)	1(7.1)	0(0.0)	0(0.0)	14(100.0)
	New	19(38.8)	13(26.5)	11(22.4)	1(2.0)	4(8.2)	1(2.0)	49(100.0)
	Sub-total	26(41.3)	17(27.0)	13(20.6)	2 (3.2)	4(6.3)	1(1.6)	63(100.0)
2021	Old	7(14.9)	15(31.9)	20(42.6)	5(10.6)	0(0.0)	0(0.0)	47(100.0)
	New	10(22.2)	18(40.0)	11(24.4)	5(11.1)	1(2.2)	0(0.0)	45(100.0)
	Sub-total	17(18.5)	33(35.9)	31(33.7)	10(10.9)	1(1.1)	0(0.0)	92(100.0)
2022	Old	9(20.9)	15(34.9)	11(25.6)	8(18.6)	0(0.0)	0(0.0)	43(100.0)
	New	14(31.8)	16 (36.4)	10 (22.7)	2(4.5)	1(2.3)	1(2.3)	44(100.0)
	Sub-total	23(26.4)	31(35.6)	21(24.1)	10(10.5)	1(1.1)	1(1.1)	87(100.0)
	**Total**	66 (100.0)	81(100.0)	65(100.0)	22(100.0)	6(100.0)	2(100.0)	242(100.0)

Evaluating the individual gynecological cancers: in 2020, out of the 49 new cases, cancer of the cervix was highest, 19 (38.8%) followed by cancer of the ovary, 13 (26.5%) and endometrial cancer, 11 (22.4%), vulva, 4 (8.2) while choriocarcinoma and cancer of the vagina were the least with 1 (2.0%) each. In 2021, ovarian cancer, had the highest occurrence, 18(40.0%) out of the 45 new cases of gynecological malignancies, followed by endometrial cancer, 11 (24.4%), the cancer of cervix was third on the rank, 10 (22.2%) while choriocarcinoma was 5 (11.1%) and the cancer of vulva, 1 (2.2%). For 2022, 44 new cases were recorded out of which ovarian cancer was also the highest, 16(36.4%) followed by cancer of cervix and then endometrium, 14(31.8%) and 10 (22.7%) respectively. Choriocarcinoma, cancers of vagina and vulva were 1 (2.3%) each. From 2020 to 2022, there was a variation in the age ranges affected which ranged between 29 years (for GTN) to 75 years in 2021 and 27 years to 81 years in 2022. Cervical cancer occurred as early as 24 years of age contrary to expectations. Considering the age distribution of the affected patients generally, 60-69 years were affected most, 21 (33.3%), followed by 40-49 years, 13 (20.6%) while the least affected were aged 90-99, 1 (1.6%) and those <20 years of age. The differences in the age group affected in the years of review were statistically significant (P=0.042).

## Discussion

Gynecological cancers are a major cause of cancer morbidity and mortality worldwide. At the Federal University Teaching Hospital, Owerri, (FUTHO) Nigeria, 17.6% of gynecological admissions were as a result of cancers. This is a very high proportion in a tertiary health facility considering previous reports of 11.5% at Aminu Kano Teaching Hospital (AKTH) Nigeria, between October 2008 and September 2011 [10], 5.5% at University of Abuja Teaching Hospital (UATH), Nigeria, from January 1^st^, 2014 to December 31^st^, 2018 [11], 3.6% of at Ogbomosho from January 1^st^, 2015 to December 31^st^, 2019 [12], 5.4% in Jos [13] 3.0% of gynecological disease burden in UATH (between 1^st^ January, 2012 and 31^st^ December, 2016 [9], 5.4% [14] in Abakaliki Nigeria, 8.4% between 1^st^ January 2012 to 31^st^ December 2013 [15] and 10.1% in Enugu [16], 2.8% at Ghana [6] and 4.7% in Port Harcourt Nigeria [17]. It is noteworthy that in some centres, there are temporal changes in the contribution of gynecological cancers to admissions in gynaecological wards [18]. In UATH, an increase from 3.0% between 2012 and 2016 to 5.5% from 2014 to 2018 [9,11]. Similarly, there was an increase in Abakiliki from 8.4% [15] from 1^st^ January 2012 to 31^st^ December 2013 to 13.4% in a 2013 - 2015 review [19]. The changes in time maybe ascribed to better practice, improved health seeking behavior and awareness created resulting in more persons presenting to the health facilities for their care.

The most common gynecological cancers in this study is very different from previous reports from Nigeria and other African countries. The pattern looks like that seen in the developed countries where endometrial and ovarian cancers top the list [20]. Over the three-year period cervical cancer was cumulatively taking a distant second and this is quite inconsistent with the reports from other studies in Nigeria. Only in the 2020 report, did cervical cancer admission lead, thereafter ovarian and endometrial cancer took over. This inconsistency can be alluded to a series of awareness creation efforts and screening of women in the centre, the State (Imo State) and environs for cervical cancer over the past ten years. Most of the screened patients who had pre malignant lesions have had electrosurgical procedures, cryotherapy or simple hysterectomy. Uptake of HPV vaccination has not been optimal with a few pockets of persons vaccinating their girl children and are unlikely to have immediately contributed to this decline. In Enugu Nigeria, 78% of cancer cases were cervical cancer. In fact, in that study, ovarian and endometrial cancers took a distant second and fourth positions with 8.9% and (4.1%) respectively [5]. Similarly in Ghana, in year 2000, cervical cancer was the leading cancer with 57.8% of gynecological cancers [6], at Abuja Nigeria, 52.7% [11], 60.6% at Abakiliki [15], Ilorin (59.6%) [21] and Benin Nigeria, (62.9%) [14]. In Nepal in a 2004 report, cervical cancer took the lead with 78.23 % of cases. In a recent publication in Nigeria, cervical cancer still took the lead at Ogbomosho, with 60.2% [12]. On the contrary, in a 2006 report from Pakistan histopathology centre, the ovary contributed 42.4% of gynecological malignancies followed by cervical cancer, 23.86% [22]. Similarly, in a tertiary facility, north India 47 out of 116 cancer cases reported on were due to ovarian cancer in April 2021 [20]. This is similar to our study in which ovarian cancer cumulatively contributed the most (28.9%) of cancer admission in the period of study. Ovarian and endometrial cancer are on the rise as seen in our study. The reason may not be very easy to allude but there may be some dietary changes that increase metabolic syndrome as well as early detection of cervical cancer (Premalignant lesions) therefore reducing the incidence of frank cervical cancers. Modifications in lifestyle, education and awareness could be important contributors.

Vaginal cancer appears to be an uncommon cancer in this study accounting for 4 out of the 242 cancer admissions (3.5%) during the period. However, this was higher than other studies who had did not have a single case of vaginal cancer reported over a 10 years period reviewed [5], and in Nepal [7] where vulva and vagina accounted for around 2% of cancers. Choriocarcinoma was much more prevalent in this study than in other reports in literature. It accounted for 9.2 % of cases over the study period unlike what was reported in Nepal (1%) [7]. Further studies to possibly confirm and ascertain the reason for this increase in our study will be needed. The wide age range for cancer cases of 19 to 91 years, was somewhat similar to that recorded in Enugu, eastern Nigeria in a 10 years review between 2000 and 2009 [5]. The median age for cervical cancer in our study was 55 years. This differs slightly with the mean age of 46.25 ± 4.99 years in Kano Nigeria [10] and other reports of 50.3 and 52 years in Ghana [6] and Kenya respectively [18]. The peak age range for cervical cancer varied over the years from 60 to 69 years in 2020, 50 to 59 years in 2021 and 40 to 49 years in 2022. There appeared to be a reducing trend in the age of presentation with cervical cancer. In 2020, cervical cancer was the commonest cancer in all the age brackets with the exception of the extreme age groups below 20 and above 90 years of age. The mean age for all the cancers was 54.5 years in 2020, 55.5 years in 2021 and 50.5 years in 2022. In 2021, the most common cancer was ovarian cancer and this occurred in the 50-59 age bracket, whereas endometrial cancer topped the 60 to 69 age bracket. All the major sites (cervix, ovary, endometrium and choriocarcinoma occurred in the 20 to 29 age bracket in equal proportions. No cancer was detected at the extremes of age of 19 and below in 2021. The age range with the highest proportion of cancer was the 40 to 49 years´ age bracket in 2022 but 60 to 69 age bracket for 2021 and 2020.

**Limitations:** our study was retrospective in design and thus it could be possible to have missed some data of patients who may not have been properly recorded by the hospital staff involved.

## Conclusion

The incidence of cervical cancer tends to be on the decline in the years reviewed while that of ovarian and endometrial were on the rise. This report shows a possible change in lifestyle and improved access to cervical cancer prevention strategies. It implies that, if the communities in Nigeria will have a sustainable cervical cancer prevention program and not just periodic screening by non-governmental agencies, the 2030 strategies and goal of the World Health Assembly can be achievable. Unfortunately, there is no established routine screening method for ovarian and endometrial cancers and therefore they will be on an apparent increase. It is also assumed that all the facilities that have recorded cervical cancer as the most common cancer can actually have a similar result as ours if a more current review is done. This is reassuring and encourages practitioners to keep embarking on prevention programs and reporting the outcomes periodically.

### What is known about this topic

- Cervical cancer ranks first in the prevalence of gynaecological cancers in African countries unlike in most developed countries where endometrial and ovarian take the lead;
- Strategized screening and prompt treatment of premalignant cervical lesions can significantly reduce the prevalence of cervical cancer;
- Childhood vaccination against the causative agent of cervical cancer is a confirmed preventive measure but the uptake in African countries including Nigeria is still very low.

### What this study adds

- Ovarian and Endometrial cancers now top the rank in our hospital;
- Implementation of evidence-based protocols in screening and treatment of premalignant and early stages of cervical cancers in our hospital over the years could have contributed to this finding.
